# Understanding Host Immunity and the Gut Microbiota Inspires the New Development of Vaccines and Adjuvants

**DOI:** 10.3390/pharmaceutics13020163

**Published:** 2021-01-26

**Authors:** Kyosuke Yakabe, Jun Uchiyama, Masahiro Akiyama, Yun-Gi Kim

**Affiliations:** 1 Research Center for Drug Discovery, Faculty of Pharmacy, Keio University, Tokyo 105-8512, Japan; kkkeeoosuyi@keio.jp (K.Y.); jun.uchiyama6127@keio.jp (J.U.); akiyama.masahiro@keio.jp (M.A.); 2 Division of Biochemistry, Faculty of Pharmacy, Keio University, Tokyo 105-8512, Japan; 3 Environmental Biology Laboratory, Faculty of Medicine, University of Tsukuba, Ibaraki 305-8575, Japan

**Keywords:** infection, vaccine, adjuvant, immunity, trained innate immunity, gut microbiota, prebiotics, probiotics

## Abstract

Vaccinations improve the mortality and morbidity rates associated with several infections through the generation of antigen-specific immune responses. Adjuvants are often used together with vaccines to improve immunogenicity. However, the immune responses induced by most on-going vaccines and adjuvants approved for human use vary in individuals; this is a limitation that must be overcome to improve vaccine efficacy. Several reports have indicated that the symbiotic bacteria, particularly the gut microbiota, impact vaccine-mediated antigen-specific immune responses and promote the induction of nonspecific responses via the “training” of innate immune cells. Therefore, the interaction between gut microbiota and innate immune cells should be considered to ensure the optimal immunogenicity of vaccines and adjuvants. In this review, we first introduce the current knowledge on the immunological mechanisms of vaccines and adjuvants. Subsequently, we discuss how the gut microbiota influences immunity and highlight the relationship between gut microbes and trained innate immunity, vaccines, and adjuvants. Understanding these complex interactions will provide insights into novel vaccine approaches centered on the gut microbiota.

## 1. Introduction

Humans, the same as all other living organisms, are subject to infection. Emerging pathogens have the potential to cause pandemic events, such as the ones caused by the *Yersinia pestis* infection in the 14th century (causing an estimated 50 million deaths, approximately half of them in Asia and Africa and the other half in Europe), influenza virus infection in the beginning of the 20th century (causing an estimated at least 50 million deaths worldwide), and SARS-CoV-2 infection (COVID-19) in the present day (causing about 2 million confirmed deaths worldwide) [1,2,3]. These pathogens have threatened our lives and resulted in a lot of deaths worldwide. However, it is possible to counter through the development of vaccines. The first human vaccine candidate was introduced by Edward Jenner in the 18th century [4], and as a direct consequence, smallpox was successfully eradicated more than 40 years ago. Thereafter, many vaccines have been established, such as the Bacille Calmette–Guerin vaccine, the pneumococcal vaccine, the diphtheria-tetanus-pertussis vaccine, the measles vaccine, and the trivalent influenza vaccine, among others. These vaccines have improved the mortality and morbidity rates associated with many infectious diseases; however, two major problems remain: (1) most antigens, alone, have a less-than-optimal immunogenic potential [5], and (2) not all of the vaccinated individuals can mount appropriate antigen-specific immune responses [6]. Therefore, to improve the immunogenicity of vaccines, adjuvants are often used; however, there are few types of adjuvants that are considered safe for human use and can induce the required immune responses. Therefore, it is essential to develop more innovative vaccine approaches and adjuvants.

Contrary to pathogenic bacteria, symbiotic bacteria have established mutual relationships with their hosts for a long time [7]. In particular, the gastrointestinal tract has a huge, complex, and diverse community of microorganisms, termed “gut microbiota”. Since, the gut microbiota significantly affects host immunity, gut microbe-associated molecules might become new potent adjuvants.

In this review, we first describe current vaccines against several respiratory tract infections, which often cause more severe consequences; we also discuss the mechanisms of clinically or experimentally used adjuvants, focusing on their impacts on the host immune systems. Finally, we focus on the interaction between innate immune cells and gut microbes. Since it is more and more accepted that the gut microbiota educates and trains innate immune cells to mount optimal immune responses against pathogens, the understanding of such mutual interactions may contribute to the development of new vaccine approaches or adjuvants in the future.

## 2. The Effect of Vaccination on Our Immune Systems

Vaccines confer individuals the resistance against specific pathogens by eliciting antigen-specific antibody/cell responses and immune memory. However, although the concept of vaccines is simple, there are several complex factors to consider for the development of effective vaccines, translating into questions such as (1) what is the best route of administration (e.g., mucosal, subcutaneous, or intramuscular)? (2) What is the immune response required to do for the elimination of pathogens? (3) How can we stimulate adaptive immunity effectively? In short, it is important to understand host immunity and the features of pathogens and infections well.

Infections can take place at every part of our bodies, including the respiratory tract, gastrointestinal tract, urinary tract, and skin; however, infections of the respiratory tract often result in more severe consequences [8]. Therefore, in this review, we particularly focused on respiratory infections, such as tuberculosis, pneumococcal pneumonia, and influenza virus infections. Below, we introduce the epidemiology and features of these particular diseases/infections and subsequently discuss the respective vaccines available and in development, as well as their mechanisms of action (Table 1).

### 2.1. Tuberculosis and Bacille Calmette Guerin (BCG) Vaccine

Tuberculosis (TB) is a contagious infectious disease that mainly affects our lungs, but can also spread to other parts of our bodies. TB is caused by *Mycobacterium tuberculosis*, an intracellular pathogen that can either cause active disease or remain latent. According to the World Health Organization (WHO), 10 million people are infected with and 1.5 million people die from TB in the world every year [9]. The mortality and mobility rates of TB have decreased gradually; however, TB remains to be one of the major public health threats along with, for instance, human immunodeficiency virus (HIV) infection [10]. *M. tuberculosis* is transmitted by airborne infectious aerosol particles and is able to reside in many types of cells, including macrophages, epithelial cells, and adipocytes after the invasion into our bodies [11]. In this way, *M. tuberculosis* escapes from host immunity and is able to survives during the latency phase. Thus, to eradicate *M. tuberculosis*, it is important to induce cellular responses to directly kill *M. tuberculosis* bacteria or cytotoxic responses to *M. tuberculosis*-infected cells [12]. In fact, type 1 cytokines, such as IFN-γ and IL-12p40, and inflammatory cytokines, such as TNF-α, are essential for the control of *M. tuberculosis* infection; this notion was proved by several studies showing that cytokine-deficient mice succumbed more to *M. tuberculosis* infection than the respective littermate controls [13,14,15]. Importantly, these type 1 cytokines are mainly produced by innate immune cells and type 1 helper T (Th1) cells, the latter considered as the most important cells for the control of TB. In agreement with this statement, clinical observations suggested that the HIV-induced deficiency in CD4^+^ T cells leads to susceptibility to TB and the reactivation of latent *M. tuberculosis* infection [16]. Therefore, vaccines to prevent *M. tuberculosis* infection, in particular, should be developed to effectively induce Th1 cells.

Bacille Calmette–Guerin (BCG) vaccine, consisting of a live-attenuated *M. bovis* strain, was first introduced in 1921, and is the only currently available vaccine against TB [17]. The BCG vaccine is administered via the intradermal route and was shown to induce IFN-γ^+^ CD4^+^ T cells (Th1 cells) in human neonates [18]; furthermore, the BCG vaccine was also reported to increase proinflammatory T cells such as IFN-γ^+^ CD8^+^, TNF-α^+^ or IL-17A^+^ CD4^+^ T cells, and especially polyfunctional CD4^+^ T cells (IFN-γ^+^, TNF-α^+^, and IL-2^+^) in the peripheral blood of the healthy adults and infants [19,20]. As mentioned before, IFN-γ and TNF-α are essential for the control of the TB pathology; however, it was also implied that IL-17, produced by Th17 cells, and other Th17-associated cytokines, such as IL-22 and IL-23, are equally needed to prevent the development of TB. Indeed, as per clinical observations, the frequency of Th17 cells, and the levels of IL-17A in active TB patients are significantly lower than those in healthy controls and latent TB individuals [21,22]. IL-17 induces the expression of the chemokines CXCL9, CXCL10, and CXCL11, facilitating the pulmonary recruitment of Th1 cells [23]. Additionally, IL-17AR mediates the expression of CXCL1 and CXCL5, which are important for the recruitment of neutrophils into the lungs of *M. tuberculosis*-infected mice [24]. However, there are studies that support that IL-17 and IL-22 may not be necessary for the prevention of TB; for instance, wild type mice administered anti-IL-17A antibodies, as well as both IL-17RA and IL-22 KO mice, are not more susceptible to *M. tuberculosis* infection than the respective controls [25].

At least 15 vaccine candidates have been developed within the last decade in the context of TB. However, although they were shown to markedly elicit antigen-specific IFN-γ producing Th1 cells, none of them was proven to prevent TB more effectively than the BCG vaccine [26]. Therefore, it is suggested that inducing antigen-specific IFN-γ producing Th1 cells is necessary but not sufficient to achieve protection against TB. Other immune cells, such as Th17 cells, CD8^+^ cytotoxic T lymphocytes (CTLs), and phagocytes, are most likely equally important, and enhancing these cells’ function might be required to develop an effective vaccine for TB.

### 2.2. Bacterial Infectious Pneumonia and Streptococcus pneumoniae Vaccine

Bacterial infectious pneumonia is mainly caused by *Streptococcus pneumoniae* (*S. pneumoniae*), Gram-positive and facultative anaerobic diplococcus. *S. pneumoniae* is an opportunistic pathogen that takes advantage of individuals with less-than-optimal immune responses such as young children, the elderly, and of immunocompromised, such as HIV-infected and transplanted patients [27]. In fact, the WHO reported that a child died from *S. pneumococcus* infection every 20 s around the world in 2017 [28]. *S. pneumoniae* asymptomatically colonizes the upper respiratory tract, and nasopharynx; however, in immunocompromised individuals, this pathogen can enter into the sterile tissues (e.g., lower respiratory tract, such as lungs, trachea, and bronchi), resulting in severe conditions such as pneumonia, otitis media, sinusitis, and meningitis. Indeed, more than 80% of lower respiratory tract infections worldwide are caused by *S. pneumoniae* [29]. Thus, preventing *S. pneumoniae* invasion into our bodies is the most important strategy to avoid severe systemic *S. pneumoniae* infection. There are a lot of different innate immunity-mediated protective strategies in the context of mucous, such as the mucus layer, and the secretion of antimicrobial peptides; however, here we focus on adaptive immunity since it has been revealed that CD4^+^ T cells and secretory-IgA (sIgA) play a key role in the control of *S. pneumoniae* infection in mouse and human studies [30,31]. In particular, Th17 cells are important for the clearance of *S. pneumoniae* [32]. Th17 cells release IL-17, which contributes to the recruitment and activation of macrophages, monocytes, and neutrophils to infection sites, promoting the elimination of *S. pneumoniae*. In fact, an increase in IL-17 production is associated with the reduction in *S. pneumoniae* burdens in the nasopharynx of mice and infants. Additionally, sIgA antibodies are also important for the opsonization of *S. pneumoniae* bacteria, promoting their phagocytosis by macrophages [33]. Of note, IgA-deficient patients showed lower pneumococcal vaccine responses and a higher rate of recurrence in the context of *S. pneumoniae* infection. Additionally, IgA-deficient mice were shown to allow the colonization by high levels of *S. pneumoniae*, although they had high levels of antigen-specific IgG [31,34]. Therefore, inducing Th17 cells and sIgA should be the aim of vaccines to prevent *S. pneumoniae* infection.

Thus far, at least 97 serotypes of *S. pneumoniae* have been identified and characterized [35]. The serotypes, 1, 14, 23F, 19F, 6A, and 19A are associated with invasive strains [36]. Currently, there are two types of vaccines against *S. pneumoniae*, the pneumococcal polysaccharide vaccine 23 (PPSV23) and the pneumococcal 13-valent conjugate vaccine (PCV13). The Advisory Committee on Immunization Practices (ACIP) in the United States recommends that a single dose of PPSV23 is routinely given to adults older than 65 years of age, and effective in 50–70% of individuals [37,38]. This vaccine works in a T cell-independent manner. It is composed of polysaccharides that are recognized by B cells allowing their subsequent differentiation into plasma cells that produce polysaccharide-specific antibodies [39]. However, because PPSV23 elicits serum IgG but not sIgA in the nasopharynx and leads to a less-than-optimal memory B cells’ pool, PPSV23 is not sufficient to prevent the incidence of pneumonia or morbidity [40,41]. Therefore, PCV13 was developed to overcome these limitations. In contrast, PCV13 can elicit T cell-dependent responses due to the chemical conjugation of the polysaccharides with carrier proteins, such as tetanus toxin, diphtheria toxin, and cross-reactive material 197 (CRM197; mutated diphtheria toxin) [42]. In addition to the recognition of polysaccharides, B cells are able to process the carrier proteins and present the resulting peptides to T cells in association with MHC-II molecule, which leads to long-lasting immune memory [39].

Remarkably, the use of PCV13 may lead to the reduction in pneumonia cases in young children by more than 90% [38]. However, this vaccine is not without limitations. First, PCV13 only targets the 13 *S. pneumoniae* serotypes more commonly associated with infections (from the 97 serotypes identified to date). Therefore, PCV13 does not prevent the colonization by the many *S. pneumoniae* serotypes not contemplated in the vaccine formulation. Another concern is that 3–19% of pneumococcal disease is caused by nonencapsulated *S. pneumoniae* [35]. As stated previously, both PCV13 and PPSV23 are composed of polysaccharides (parts of the capsule of *S. pneumoniae*) as antigens, and therefore, they do not confer protection against nonencapsulated *S. pneumoniae*. These limitations should be considered in the development of future vaccines against *S. pneumoniae* with a broader spectrum.

### 2.3. Influenza Virus Infection and Vaccine

Seasonal influenza is an acute respiratory infection caused by influenza viruses. Worldwide, annual epidemics are estimated to result in about 3 to 5 million cases of severe illness, and 290,000 to 650,000 respiratory deaths [43]. Although most people develop a mild condition with fever and other minor symptoms, influenza virus infection can cause more severe respiratory disorders and even death, especially in young children, the elderly, pregnant women, and immunocompromised individuals [43]. The influenza virus is part of the *Orthomyxoviridae* family, composed of enveloped viruses with negative-sense single-stranded RNA; of note, influenza viruses are classified into 4 types, A, B, C, and D [44]. Influenza A viruses are further classified into subtypes based on their surface glycoproteins, hemagglutinin (HA), and neuraminidase (NA). Seasonal influenza is mainly caused by influenza A, H1N1, and H3N2 subtypes, and by influenza B. Recurrent influenza epidemics occur every year despite the pre-existing immunity because influenza viruses escape immune recognition using strategies such as antigenic drift and antigenic shift, gradually accumulating point mutations on HA and NA [45].

Influenza virus infections are initiated at the mucosal sites of the respiratory tract. Influenza virus invades into host cells through the direct binding of HA to cell surface molecules (sialic acid) and the consequent endocytosis. Thus, high-affinity mucosal sIgA is important to prevent the invasion of the influenza viruses [46]. Moreover, systemic IgG is able to bind to HA, and NA and inhibit the attachment to or release from host cells directly. Additionally, as an indirect mechanism, IgG mediates antibody-dependent cell-mediated cytotoxicity (ADCC) and antibody-dependent cellular phagocytosis (ADCP) through the Fc receptor (FcR) [47,48]. However, the influenza virus undergoes significant antigenic variation, two types of antigenic variation: antigenic drift and genetic shift, that elicits viral escape from neutralization by antibodies generated in prior vaccinations. Therefore, the development of a universal influenza vaccine is required to protect the host from antigenic variation, and to confer lifelong immunity.

There are two types of influenza vaccines currently available: live-attenuated influenza vaccines (LAIVs) and inactivated influenza vaccines (IIVs; split or subunit vaccines), including the influenza A H1N1 and H3N2, and influenza B strains. Interestingly, a previous study revealed that intranasal LAIV inoculation but not intraperitoneal IIV injection led to the establishment of CD4^+^ or CD8^+^ long-lived resident memory T (T_RM_) cells; IIV only promoted the neutralizing antibody responses [49]. Importantly, nasal CD8^+^ T_RM_ cells play a crucial role in the protection against the influenza virus infection [50]; in fact, nasal CD8^+^ T_RM_ cells develop independently of local cognate antigen recognition, representing a long-term protective population. In some instances, the vaccine-mediated induction of virus-specific T_RM_ cells provided heterosubtypic protection against other influenza virus strains (not contained in the vaccine). This result suggests that the induction of T_RM_ cells is very important to achieve protection against heterogeneous influenza virus strains.

Therefore, the development of a universal influenza vaccine that confers protection against multiple influenza virus subtypes is both quite needed and not impossible, if we focus (1) on conserved antigenic domains and/or (2) on the induction of CTLs, which are able to kill virus-infected cells. In fact, a vaccine targeting the influenza virus conserved domain M2e was already proposed [51]. However, after immunization, the M2e-specific antibody titers steadily declined over 1 year [52,53]. Therefore, there is still a lot of room for improvement.

## 3. Adjuvants

Most of the currently available vaccines have lower immunostimulatory potencies (compared with live virulent pathogens) because they are inactivated or attenuated pathogens or consist of pathogen components to avoid severe side effects. Therefore, adjuvants are extremely important in vaccine design, to enhance the immune responses to vaccines by various mechanisms. For instance, adjuvants are used to (1) promote the antigen-specific antibody responses effectively, especially in the elderly and infants who have a lower capability to mount an immune response to vaccines, and to (2) facilitate the use of smaller doses of antigens [5]. However, although adjuvants are used in humans and animals, their cellular and molecular mechanisms are not fully understood. Here, we review the immunological mechanism of the different adjuvants commonly used as well as of those that were recently discovered/approved to reference more recently published studies (Table 2).

### 3.1. Alum

Aluminum hydrates (alums) are the most commonly used adjuvant in humans and animal experiments. Alum elicits Th2 responses, promoting the production of IgG1 and IgE, but not cell-mediated immunity (Th1 responses) and cytotoxic T cells [54]. Alum was initially thought to promote the formation of a long-lasting depot of antigens, promoting their uptake by antigen-presenting cells (APCs) [55]; of note, our understanding of alum has greatly improved with the advance of studies in innate immunity. For instance, in 2008, several studies indicated that alum-activated caspase-1 through the canonical inflammasome pathway (NOD-like receptor (NLR) family pyrin domain containing 3-NLRP3—protein-mediated), resulting in the release of IL-1β [56,57,58,59]. Additionally, it was suggested that uric acid, which is a major metabolite of nucleic acids and one of the damage-associated molecular patterns (DAMPs) released from dying cells, is required for alum-induced Th2 responses [60]. Uric acid was also shown to activate the NLRP3 inflammasome and induce the secretion of IL-1β production by macrophages and neutrophils [61,62], which in turn promoted the recruitment of inflammatory (migratory) dendritic cells (DCs) into the inflammatory site. Inflammatory DCs are involved in the induction of Th2 responses (secretion of IgG1, IL-4, and IL-5) in the context of alum. Altogether, these reports suggested that dying host cells’ release of uric acid after alum injection, contribute to the induction of Th2 responses through NLRP3-mediated inflammation.

However, in 2011, Marichal et al. showed that both NLRP3- and caspase1-deficient mice were able to develop a humoral immune responses comparable to those in wild-type (WT) mice after alum injection [63]. Furthermore, these authors revealed that host DNA is released from dying cells at the injection site, acting as DAMPs and promoting endogenous immunostimulatory signals in the context of alum. This alum-associated host DNA release was shown to promote the production of antigen-specific IgE and IgG1 either via the IRF3-Tbk1 axis or in an IRF3-independent manner, respectively. There are some DNA-sensing molecules upstream of the IRF3-Tbk1 axis; however, it remains unknown which of these molecules is essential for IgE induction in the context of alum. We only know that MAVS and ZBP1 are not involved since *Mavs*^−/−^ and *Zbp1*^−/−^ mice did not show reduced antigen-specific IgE or IgG1 production in the context of alum [63]. Although the detailed mechanism behind the adjuvant activity of alum remains unknown, these studies have impacted the development of DNA vaccines.

### 3.2. Complete Freund’s Adjuvant

Complete Freund’s adjuvant (CFA) is usually used in animal models to emulsify aqueous antigen solutions and immunize with antigens. CFA is composed of paraffin oil (incomplete Freund’s adjuvant: IFA) and a surfactant with heat-killed *M. tuberculosis* or *M. butyricum* [5]; the mycobacterial peptidoglycan and trehalose dimycolate act as the immunostimulatory components. Because immunization with CFA induces strong Th1 and Th17 immune responses, CFA is often used in T cell-dependent contexts, including the collagen-induced arthritis and experimental autoimmune encephalomyelitis mice model [64,65]. The peptidoglycan contained in CFA is sensed by Toll-like receptor (TLR) 2 and the nucleotide-binding oligomerization domain containing (NOD) 1, but TLR2 is not necessary for inducing an antigen-specific immune responses in the context of CFA, supported by the fact that *Tlr2*^−/−^ mice showed the same levels of pathogenesis compared with *Trl2*^+/+^ littermates in experimental autoimmune uveitis induced by CFA [66,67]. On the other hand, NOD1, a cytosolic pattern-recognition receptor (PRR) expressed ubiquitously, is required to the antigen-specific immune responses in the context of CFA [68]; NOD1-deficient mice showed the downregulation of inflammatory cytokine production, such as TNF-α, IL-1β, IL-6, and IL-12p40 in DCs, which consequently compromised the development of antigen-specific adaptive immunity.

Additionally, the second mycobacterial component in CFA, trehalose dimycolate (TDM), is recognized by Mincle, a C-type lectin receptor (CLR) which signals through the Syk kinase-CARD9 pathway, resulting in the secretion of inflammatory cytokines, IL-1β and IL-6 [69,70]. These inflammatory cytokines are involved in the Th1/Th17 polarization of helper T cells. Th1 differentiation is regulated by the expression of the transcription factor, T-bet, which is dependent on IL-12 and signal transducer and activator of transcription 4 (STAT4) [71,72,73]. Th17 cell differentiation is regulated by the RAR-related orphan receptor gamma (RORγt) expression. Transforming growth factor-β (TGF-β) and IL-6 synergistically induce Th17 cells in vitro [74]; additionally, it was also reported that the IL-1/IL-1R axis is important in Th17 cell differentiation in the context of mice immunized with CFA. For instance, one report suggested that the NLRP3 inflammasome-mediated IL-1β production is involved in the Th17 differentiation of Th17 cells, independently of NOD1/2 [66]. This report argued that the TDM-Mincle-Syk-CARD9 signaling pathway plays a major role in IL-1β production. Indeed, the Th17-promoting activity of CFA was compromised in caspase-1- and CARD9-deficient mice. Additionally, the other report showed that IL-1 signaling via IL-1R1 induced IRF4 and RORγt expression during early Th17 polarization [75]; IL-1 synergizes with IL-6 to regulate Th17 differentiation. Moreover, the IL-1α-IL-1R1 axis is also required for the secretion of CFA-induced cytokines from Th17 cells [76]. However, the contribution of IL-1α versus IL-1β in the generation of Th17 response remains unclear.

In summary, immunization with CFA elicits Th1 and Th17 cells via the production of inflammatory cytokines by innate immune cells. NOD1 and Mincle are involved in the secretion of inflammatory cytokines production and, consequently, in the subsequent induction of adaptive immune responses.

### 3.3. Cholera Toxin (CT)

Cholera toxin (CT), an enterotoxin secreted by *Vibrio cholerae*, the causative agent of cholera-associated diarrhea, is one of the mucosal adjuvants often used in animal experiments. CT is composed of one A catalytic subunit that transfers the ADP-ribose moiety from NAD^+^ to G_sα_, stimulating the production of cyclic AMP by adenylate cyclase, and five B subunits, GM1 gangliosides that interact with glycosphingolipids on the surface of mammalian cells [77]. CT can induce antigen-specific immune responses when it is administered by the nasal or oral routes [78,79]. Previously, CT was known as a type 2 immunity-inducing adjuvant as the upregulation of IL-4 and IL-5, and IgG1 and the downregulation of IL-2 and IFN-γ have often been reported [80,81]. However, different studies showed that intracellular cAMP and G_sα_ positively regulate Th1 and Th17 immune responses [79,82]. Therefore, CT-induced immune responses remain controversial.

Kim et al. studied the adjuvant mechanism of CT in detail; they showed that NOD2 and the serine-threonine protein kinase Ripk2 (the adaptor required for NOD signaling), but not NOD1, are required for the occurrence of antigen-specific immune responses in the context of CT [83]. Moreover, they revealed that the symbiotic bacteria are also involved in the development of antigen-specific immune responses after the nasal administration of CT. Symbiotic bacteria are sensed by host PRRs, including TLRs and NLRs, and trigger innate immune responses [84,85,86]. In fact, it was argued that bacteria in the nasal cavity of mice, including *Staphylococcus sciuri* and *Bacillus clausii*, contribute to the CT-mediated increase in antigen-specific IgG production in a NOD2/CD11c^+^ cell-dependent manner. The adjuvanticity of CT was not abrogated in MyD88^−/−^ mouse, suggesting that the effect of CT is not dependent on TLR signaling. Furthermore, the necessity of NOD2 was also observed in the context of oral CT administration [87]. This report further supports the importance of the microbe–NOD2 axis and indicates that IL-1β is required for the adjuvant activity of CT. IL-1β produced by DCs dictates the differentiation of CD4^+^ T cells into Tfh cells [88]; in turn, Tfh cells support the proliferation and activation of B cells through germinal center reactions [89]. Additionally, Yang et al. showed that gut microbiota-derived short-chain fatty acids (SCFAs), especially acetate and butyrate enhanced antigen-specific IgA and IgG productions elicited by the administration of CT through G-protein coupled receptor 43 (GPR43). In vitro experiments further showed that both acetate and butyrate treatments upregulated the expression of BAFF and ALDH1a2 in DCs; these molecules induce the expression of IRF4, Blimp1, and XBP4, which are crucial for the differentiation of B cells into plasma cells [90].

Taken together, these reports suggest that NOD2 and Ripk2 signaling is essential to the adjuvant potential of CT and that symbiotic microbes regulate CT adjuvanticity through their microbial components and metabolite sensing.

**Table 2:** The different types of adjuvants and their immunological mechanisms.

Adjuvants	Components	Activated Immune Responses	Mechanism
Alum	Aluminum hydroxide	・Th2 response ・IgG1 and IgE production	・DNA released by dying cells is sensed as DAMPs. ・IgE is elicited by IRF3-Tbk1 axis, while IgG1 is an IRF3-independent pathway [63].
Complete Freund’s adjuvant (CFA)	Heat-killed *M. butyricum* (peptidoglycan and trehalose-6,6′-dimycolate (TDM))	・Th1 and Th17 response ・Cellular immunity	・TDM-Mincle-Syk-CARD9 and peptidoglycan-Nod1 signaling pathways are involved in inflammatory cytokine production [69,70]. ・Th1: IL-12-STAT4 axis regulates T-bet expression [71,72,73]. ・Th17: IL-1-IL-1R axis regulates RORγt and IRF4 expression [75].
Cholera toxin	Enterotoxin secreted by *Vibrio cholerae*	・Th2-associated cytokines, IL-4, IL-5, and IgG1 production ・Upregulating Th1 and Th17 responses	・Nod2, Ripk2, and IL-1β are required for CT adjuvanticity [83]. ・Symbiotic bacteria (*Streptococcus sciuri* and *Bacillus clausii*) and SCFAs contribute to enhance antigen-specific IgG production by CT [83,90]. ・IL-1β dictates CD4^+^ T cells to differentiation of Tfh cells, which support antibody production from B cells [88].
Pulmonary surfactant-biomimetic liposomes encapsulating 2′, 3′-cyclic guanosine monophosphate adenosine monophosphate (PS-GAMP)	Liposome ・Dipalmitoylated phosphatidylcholine (DPPC) ・Phosphatidylglycerol (DPPG) ・Cholesterol ・Polyethylene glycol (PEG) (Trehalose lyophilization) Content ・2′, 3′-cyclic guanosine monophosphate adenosine monophosphate (cGAMP)	・Enhance intrasubtype-specific IgG and IgA production. ・Induce high frequency of CD8^+^ resident memory T (T_RM_) cells. ・Activating CD8^+^ Granzyme B^+^ T cells	・Surfactant proteins, SP-A and SP-D, bound to PS-GAMP, and subsequently, PS-GAMP is caught by alveolar macrophages (AMs). ・cGAMP is released in cytoplasm and stimulates STING in AMs and type 2 alveolar epithelial cells (AEC II), resulting in secreting IFN-α/β and GM-CSF. ・Secreted cytokines activate DCs, which subsequently induce adaptive immunity, CD8^+^ T cells activation, and immunoglobulin production [91].

### 3.4. PS-GAMP

In 2020, Wang et al. reported a new candidate adjuvant for use in the context of the universal influenza vaccine [91]. Pulmonary surfactant (PS)-biomimetic liposomes encapsulating 2′, 3′-cyclic guanosine monophosphate adenosine monophosphate (GAMP) is an agonist of the interferon gene inducer stimulator of interferon genes (STING). PS is composed of a unique phospholipid, termed dipalmitoylphosphatidylcholine, and four surfactant-associated proteins, SP-A, -B, -C, and -D [92]; SP-A and SP-D are required for the adjuvanticity of PS-GAMP. Importantly, the cGAMP contained within PS-GAMP was shown to stimulate alveolar macrophages and alveolar epithelial cells type II (AEC II) to secrete type 1 IFN through the STING pathways, resulting in the activation of DCs and CD8^+^ T cells. Remarkably, a PS-GAMP-conjugated influenza vaccine, induced protection against a broad range of influenza variants in mouse and ferret models; the administration of a single variant-containing vaccine and PS-GAMP led to the intrasubtype-specific IgG and IgA production, the activation of CD8^+^ CTLs, and high frequency of CD8^+^ T_RM_ cells in the lungs. While the current influenza vaccines require at least 10 to 14 days to be effective, protection was mediated only 2 days after immunization and maintained for 6 months in animals vaccinated with the influenza vaccine adjuvanted with PS-GAMP. This early protection would be the most effective means to confine viral spreading during an epidemic phase and prevent pandemics, potentially saving millions of lives.

However, how the administration of PS-GAMP can induce such early, strong, and long-term immune responses remains unclear. Therefore, the mechanism behind the adjuvant effect of PS-GAMP must be studied and disclosed. This new adjuvant has the potential to impact the development of other vaccines in the future.

## 4. Trained Immunity

The notion that immunological memory in vertebrates was established in only in the context of adaptive immune cells, such as T and B cells, was a well-established dogma. However, recent studies revealed that innate immune cells, including NK cells, macrophages, and monocytes, also showed memory characteristics. Activated innate immune cells are able to respond in an enhanced manner to subsequent triggers, today known as “trained (innate) immunity”. Briefly, the concept of trained immunity is described as the “long-term functional reprogramming of innate immune cells, evoked by exogenous or endogenous insults and which leads to an altered response towards a second challenge after the return to a non-activated state” [93]. As mentioned above, trained immunity can be evoked by the bacterial components including lipopolysaccharides (LPSs), β-glucans, or by vaccines, e.g., the BCG vaccine, which induce the epigenetic reprogramming of transcriptional pathways. Interestingly, LPSs and β-glucans induce contrary reactions in the context of monocytes; a low dose of LPSs failed to induce active histone marks in the promoters and enhancers of genes involved in the lipid metabolism and phagocytic pathways, resulting in the lower inflammatory response of monocytes to subsequent stimulation—this is known as “LPS-tolerance” [94]. In contrast, β-glucans partially reversed LPS-tolerance in vitro training monocytes to upregulate the production of inflammatory cytokines through the β-glucan receptor dectin-1 and noncanonical Raf-1 signaling [94,95]. The epigenetic change in monocytes stimulated by β-glucans also led to the upregulation of glycolysis-related genes, the elevation of aerobic glycolysis, and an increase in glucose consumption and lactate production, via the dectin-1-Akt-mTOR-HIF-1α pathway [96]. This aerobic glycolysis-dependent metabolism state is also observed in activated T cells [97]. This metabolism change is confirmed in monocytes which are trained by BCG vaccination, which is reflected by the effects on the histone marks (H3K4me3 and H3K9me3) [98].

It is not unreasonable to ask why trained immunity in monocytes can last for long periods of time taking into consideration their relatively short lifespans. Kaufmann et al. and Mitroulis et al. have provided an answer to this question. They showed that both BCG and β-glucan impact hematopoietic stem cells (HSCs), contributing to long-term trained immunity [99,100]. In fact, BCG administered intravenously (but not subcutaneously) led to the expansion of LKS^+^, characterized as Lin^−^ c-kit^+^ Sca1^+^, bone-marrow cells (HSCs and multipotent progenitors (MPPs)); importantly, their transcriptional landscape was altered, and their response to *M. tuberculosis* infection was increased. This BCG-induced protective capacity of HSCs is sustainable and β-glucan was also demonstrated to elicit the myelopoiesis of LKS^+^ cells in the bone marrow, lasting for 12 weeks (or more). However, β-glucan acts predominantly on long-term HSCs (LT-HSCs), enhancing glycolysis. These epigenetic changes in HSCs are transmitted to their progeny, resulting in a favorable response to future infections.

Because trained immunity is characterized by the epigenetic changes and not gene recombination, trained immunity leads to non-antigen-specific reactions to unvaccinated antigens (cross-reaction). Indeed, although the BCG vaccine contains *M. bovis* as an antigen, BCG vaccination can protect mice not only against *M. tuberculosis* but also other *Mycobacterial* infections [101]. Furthermore, BCG vaccination induces epigenetic reprogramming in human monocytes in vivo and confers protection against experimental infection with an attenuated yellow fever virus, with a key role of IL-1β as a mediator of trained immunity [98]. Remarkably, epidemiological data also suggest that measles, vaccinia, and diphtheria-tetanus-pertussis (DTP) vaccines also induce nonspecific reactions [102]. However, no experimental data exist in this regard; therefore, the mechanism behind such cross-reaction in the context of measles, vaccinia, and DTP vaccines still needs to be unveiled.

All of the above highlights the potential of the exploitation of trained immunity for the development of new vaccines against several infections in the future. However, the following must be considered: the antigen nonspecific responses mediated by reprogrammed innate immune cells will be higher in magnitude, with the potential to lead to harmful results, such as autoimmune diseases [103].

## 5. The Gut Microbiota and Vaccine (Adjuvant) Efficacy

### 5.1. The Gut Microbiota and Immunity

Symbiotic microorganisms reside in human bodies, including in the gut, skin, and other mucosal environments. In particular, in the gut, microorganisms shape a complex, diverse, and a huge community, collectively termed gut microbiota [104]. The human gut microbiota is mainly dominated by Bacteroidetes and Firmicutes, followed by Actinobacteria, Proteobacteria, and Verrucomicrobia at the phylum levels. Gut microorganisms contribute to a broad range of essential and beneficial functions, such as the control of mucosal immunity, the metabolization of nutrients, and the prevention of colonization by pathogens [105,106]. A healthy gut microbiota is maintained by the complex relationships between bacteria and the host immune systems; the imbalance of this equilibrium leads not only to intestinal diseases, such as inflammatory bowel disease and colorectal cancer, but also to systemic diseases, such as allergy, atopic dermatitis, obesity, diabetes, and neuropsychiatric disorders [107,108,109,110,111,112,113].

The relationship between the gut microbiota and the immune system has been well established in studies with germ-free (GF) animals. In fact, GF mice show reduced frequencies of regulatory T (Treg) cells in the intestinal lamina propria, lower levels of sIgA, and lower numbers of colonic macrophages (with compromised function) compared with those in specific-pathogen-free (SPF) mice [114,115,116]. In turn, the defect in IgA and Treg cells induces aberrant gut microbiota composition (“dysbiosis”) in mice and humans [117,118]; intestinal macrophages are also essential for shaping of the gut microbiota composition in zebrafish [119]. All of the above suggests that the gut microbiota establishes a symbiotic relationship with host immunity.

As briefly mentioned, the gut microbiota impacts not only the development of host immune cells, but also their differentiation, and function through their components and derived metabolites [120]. For instance, gut microbes contain many microbe/pathogen-associated molecular patterns (MAMPs/PAMPs), including lipopolysaccharides (LPSs), peptidoglycan, flagellin, and bacterial CpG DNA and RNA which are sensed by host PRRs, mainly expressed on immune cells, especially APCs. TLRs and NLRs are well-known PRRs that recognize specific MAMPs/PAMPs; each of them triggers cell signaling and the consequent immune activation [84,86]. Below, we discuss the interplay between MAMPs/PAMPs and innate immune cells in greater detail.

In addition to MAMPs/PAMPs, bacterial metabolites also influence host immune systems [121]. In fact, SCFAs, end products of fermentation of dietary fibers by gut microbes, such as acetate, propionate, and butyrate, play a crucial role in host immunity. For example, acetate moderates dextran sulfate sodium (DSS)-induced colitis through GPR43, and decreases the production of inflammatory cytokines [122]. Propionate, which is mainly sensed by GPR41, leads to alterations in bone marrow hematopoiesis characterized by an increase in the precursors of macrophages and DCs; this metabolite is associated with the moderation of allergic inflammation via the GPR41-dependent impaired development of Th2 responses [123]. Additionally, gut microbe-derived butyrate was shown to induce the differentiation of Treg cells in the colonic lamina propria via the promotion of epigenetic modification (increase histone H3 acetylation at both the promoter and conserved noncoding sequence 3) in the *Foxp3* gene locus [124]. Therefore, collectively, gut microbe derivatives (components and metabolites) have a remarkable impact on host immunity through each of the many recognition receptors reported to date.

Therefore, it is not unreasonable to suggest that the gut microbiota may affect vaccine efficacy; however, how gut microbiota influences the efficacy of vaccines and adjuvants is not well understood. In this review, we will discuss the association between gut microbiota and the efficacy of vaccines and adjuvants citing the latest papers. First, we will review the clinical studies on the relationship between gut microbiota and vaccine efficacy. Subsequently, we will discuss the potential adjuvant-like role of gut microbiota in the promotion of protective effects against viral infection through the stimulation of host immunity.

### 5.2. Vaccine Efficacy and Gut Microbiota

Vaccination is an essential approach to decrease the prevalence of conventional and emerging infections. However, there is a major limitation of vaccine approaches; their efficacy of vaccines is different for each individual due to not only their age, but also to genetic, and environmental factors [6,125,126]. Although the alteration of genetic-related factors (and of the age) is not possible, the environmental factors can be changed. In this review, we will focus particularly on the gut microbiota. Several clinical and animal studies have suggested that the gut microbiota impacts vaccine efficacy.

In fact, antibiotic-treated and GF mice showed impaired antibody responses to OVA/CFA vaccination [127]. Moreover, gut dysbiosis significantly decreased the activation of CD4^+^ and CD8^+^ T cells and the frequency of CD4^+^ and CD8^+^ memory T cells in the lungs of BCG-vaccinated mice [128]. These mouse studies suggest that the gut microbiota is required to elicit appropriate immune responses to the vaccine antigens. Moreover, in humans, a high relative abundance of Actinobacteria, particularly of *Bifidobacterium longum* subsp. *infantis*, was positively correlated with vaccine-specific T cell proliferation in both oral and parenteral vaccines, such as BCG, oral polio virus, and tetanus toxin in Bangladeshi infants [129]. Another report shows that *Clostridium* cluster XI and Proteobacteria, including bacteria related to *Serratia* spp. and *Escherichia coli*, were associated with rotavirus vaccine efficacy in infant responders from Pakistan [130].

Antibiotics are a well-known gut microbiota-perturbing factor. Broad-spectrum antibiotic-treated healthy adults showed low pre-existing antibody titers with a consequent impairment in influenza virus H1N1-specific neutralization mediated by IgG1 and IgA antibodies. The gut microbiota of antibiotic-treated individuals was dominated by the Enterobacteriaceae and Streptococcaceae families. Additionally, in their blood, that a decrease in secondary bile acids was observed, particularly lithocholic acid. The perturbation of secondary bile acids metabolism is associated with the upregulation of NLRP3-related genes [131]. Taken together, the above suggests that specific bacteria impact vaccine efficacy through the modulation of host metabolites and gene expression.

In early life, the gut microbiota plays a crucial role in the development and maturation of host immune systems [132]. Most vaccines, such as rotavirus, hepatitis B virus, BCG, and PCV vaccines, are administered in early life since most of the infections cause the highest morbidity and mortality rates in newborns and children. Thus, it is also important to understand how the gut microbiota influences the vaccine efficacy in early life. The fetal gut microbiota composition is extremely affected by the type of delivery of birth (vaginal delivery versus cesarean section (C-section)) and feeding mode (breastfed versus formula-based feeding). As usual, facultative anaerobic bacteria, such as *Escherichia* and *Lactobacillus*, are the first colonizers; only after do obligate anaerobes, including *Bacteroides*, *Clostridium*, and *Bifidobacterium*, colonize the intestine. Vaginal-delivered neonates are exposed to the maternal vagina, and their microbiota is dominated by *Escherichia*, *Enterococcus*, and *Lactobacillus*; on the other hand, C-section-delivered infants have gut microbiotas dominated by *Streptococcus*, *Staphylococcus*, and *Haemophilus*, typical skin and oral microbes [133,134]. In addition, it was reported that C-section-delivered individuals show an increased risk of immune disorder-related diseases, such as ulcerative colitis, celiac disease, asthma, laryngitis, and lower respiratory tract infection, suggesting that gut colonization in the context of C-section promotes undesirable host immune responses sustained for a long time [135]. Moreover, the feeding pattern also impacts the gut microbiota composition. Several studies compared the gut microbiota of vaginal-delivered breastfed with formula-fed infants. Exclusively, breast-fed infants had elevated levels of *Lactobacillus johnsonii*, *L. gasseri*, *L. casei*, and *B. longum*; on the other hand, formula-fed infants showed increased levels of *Citrobacter* spp., *Enterbacter cloacae*, *Bilophila wadswothia*, and *Actinomyces* [133,136].

Few clinical reports discuss the relationship between neonatal gut microbiota and vaccine efficacy; however, several animal studies have revealed that the exposure to antibiotics in early life impaired the induction of antigen-specific antibody production after vaccination [127,137]. For instance, Lynn et al. showed that mice born from and breastfed by mothers undergoing antibiotics showed impaired responses to both adjuvanted and nonadjuvanted vaccines, such as influenza, BCG, and PCV vaccines. Of note, their gut microbiota dysbiosis following antibiotics exposure was characterized by the loss of Bacteroidales and overgrowth of *Akkermansia*. Therefore, these results suggest that colonization by several specific bacteria in early life is required for optimal antigen-specific antibody secretion; however, there is no strong evidence available on the particular gut microbes that affect vaccine efficacy, and, consequently, the detailed mechanism behind is unknown.

**Figure 1:**
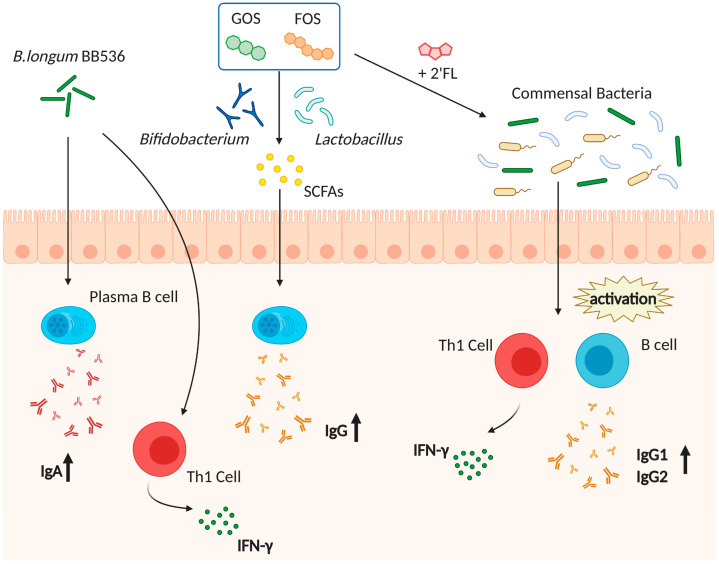
Prebiotics and probiotics interventions improve influenza virus vaccine efficacy. The use of prebiotics and probiotics improves vaccine efficacy, impacting host immune cells. As probiotics, *Bifidobacterium longum* BB536 might promote the secretion of IgA and IFN-γ. As prebiotics, galactooligosaccharide (GOS) and fructooligosaccharide (FOS), induce the increase in *Bifidobacterium* or *Lactobacillus* and the levels of short-chain fatty acids (SCFAs) in the gut, which in turn promote the increase in IgG levels. Additionally, treatment with long-chain GOS/short-chain FOS/2′-fucosyllactose (2′-FL) promotes the activation of influenza vaccine-specific Th1 and B cells, resulting in higher IgG1 and IgG2 levels via symbiotic bacteria.

To improve the gut microbiota composition, the use of prebiotics, and probiotics is proposed (Figure 1). Prebiotics are compounds that induce the growth of certain bacteria. Curiously, Vos et al. revealed that a prebiotic mixture containing fructooligosaccharide (FOS) and galactooligosaccharide (GOS) enhanced systemic immune responses to influenza virus vaccine [138]. This prebiotic intervention induced the growth of *Bifidobacterium* and *Lactobacillus* and an increase in the levels of SCFAs, suggesting that FOS/GOS-induced bacteria and metabolites might contribute to the increase in the host immune responses. Another report showed that the combination of 2′-fucosyllactose, a dominant oligosaccharide in human milk, long-chain FOS, and short-chain GOS, enhanced the activation of influenza vaccine-specific Th1 cell and B cell activation [139] and increased the levels of IgG1 and IgG2a. Moreover, Luccia et al. showed that combined prebiotics (spirulina, amaranth, and flaxseed) and microbial interventions improved the immune responses to CT vaccination in GF mice colonized with the gut microbiota of Bangladeshi nonresponder children [140]. Collectively, these results support the hypothesis that the use of prebiotics may improve vaccine efficacy.

*Lactobacillus* and *Bifidobacterium* are often used as probiotics; these bacteria species have been shown to enhance vaccine efficacy (Figure 1). *B. longum* BB536 intake for 2 weeks tended to augment serum IgA titers after trivalent influenza vaccine administration [141]. In addition, *B. longum* BB536 supplemented healthy infants showed an increased number of IFN-γ secreting cells, suggesting that *B. longum* BB536 also improves Th1 responses [142]. Additionally, the daily consumption of drinks with *L. casei* DN114-01 and *L. plantarum* CECT7315/7316 conferred higher influenza-specific antibody production after vaccination compared with the control group [143,144]. However, other reports showed that the administration of *L. casei* 431 and *L. casei* Shirota did not enhance the immune responses after influenza vaccination [145,146]. Therefore, there is controversy in the field; however, the daily consumption of probiotics seems to be required to upregulate vaccine-mediated immune responses. Further studies are needed to disclose the most appropriate strains and doses.

Altogether, the above highlights that the association between gut microbes and vaccine-induced immune responses has gradually been shown; however, it remains unknown which specific gut microbes affect vaccine efficacy, as well as the mechanism behind it.

### 5.3. Gut Microbiota Acts Similar to an Adjuvant and Protects Hosts against Several Infections through the Activation of Host Immunity

As mentioned throughout this review, the stimulation of innate immune cells, especially APCs, is required to elicit proper antigen-specific adaptive immunity and protect against infections. Furthermore, gut microbial components, including MAMPs and PAMPs, have strong immunostimulatory properties, stimulating innate immune cells through PRRs [147]. Indeed, the concept that successful antigen-specific immunity is dependent on innate immune recognition systems has been supported by the fact that the detection of MAMPs/PAMPs by PRRs provides signals for mounting antigen-specific Th1/Th2 responses [148]. In particular, the TLR-5-mediated sensing of flagellin derived from the gut microbiota was shown to affect the antibody response in influenza and inactivated polio vaccines [149]; *Trl5*^−/−^, antibiotic-treated, and GF mice were not able to produce influenza-specific IgG and IgM production as efficiently as WT mice in the context of vaccination. Remarkably, the impairment of antibody production was restored by the administration of flagellated, but not of nonflagellated *E. coli* strains. Additionally, as stated before, NOD2- but not NOD1-deficient mice cannot elicit antigen-specific antibody secretion in the context of human serum albumin and CT immunization, suggesting that NOD2 is also needed for the induction of antigen-specific immune responses [83]. Moreover, MyD88 and MyD88-mediated signaling pathways are also important for the induction of primary and boost immune responses [150]; in fact, *MyD88*^−/−^ mice showed a lower production of IgG2a/c and lower numbers of recall memory B cells versus the littermate controls in the context of influenza vaccine. Additionally, TRIF, which is also a molecule that mediates TRL signaling, was shown to contribute to the differentiation of Tfh cells, the formation of germinal centers, and the production of antibodies, through downstream IRF3- and inflammasome-mediated signaling in mice were vaccinated with live Gram-negative bacteria [88]. Altogether, these reports suggest that MAMPs/PAMPs derived from commensal microbes act as endogenous adjuvants and that host innate immune sensors and associated molecules are also needed for the mounting of proper immune responses to vaccines.

**Figure 2:**
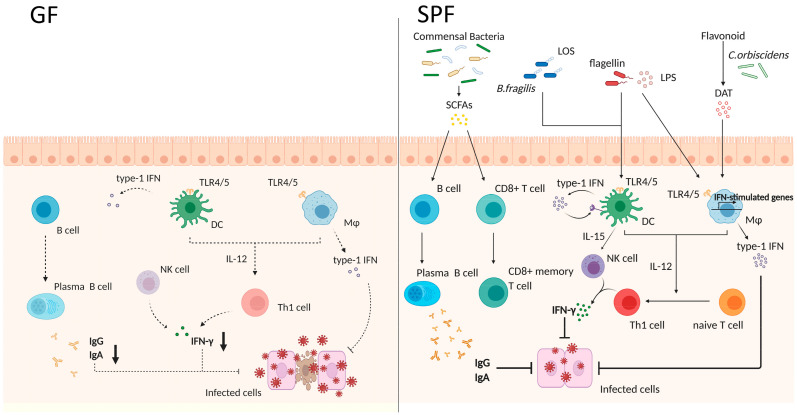
The gut microbiota is required for optimal innate immunity-mediated immune responses. The left panel shows the intestinal environment of germ-free (GF) mice. Because GF mice have no commensal bacteria, intestinal innate immune cells are less stimulated, and, consequently, secrete lower levels of cytokines. Moreover, B cells fail to differentiate into plasma cells, resulting in lower antibody secretion (IgG and IgA). The lower activity of immune cells renders the animals more susceptible to viral infection. On the other hand, in the right panel, specific-pathogen-free (SPF) mice harbor a lot of commensals, which affect host immune cells through microbe-derived components and metabolites. Microbe-associated molecular patterns (MAMPs) such as lipopolysaccharide (LPS), flagellin, and lipooligosaccharide (LOS) are recognized by pattern recognition receptors (PRRs), expressed by macrophages (Mø) and dendritic cells (DCs). These antigen-presenting cells produce type 1 IFN, and subsequently stimulate natural killer (NK) cells. NK cells induce the differentiation of naïve T cells into Th1 cells, which secrete IFN-γ and control virus infection. The microbial metabolite desaminotyrosine (DAT) produced by *Clostridium orbiscindens* also enhances type 1 IFN production. Additionally, short-chain fatty acids (SCFAs) produced by microbes promote the differentiation of B cells and activated-CD8^+^ T cells into plasma cells and memory T cells, respectively. Last but not least, plasma cells secrete IgG and IgA, rendering the animals resistant to viral infection.

There are more and more pieces of evidence showing that exposure to microbes is required to mount both optimal innate and adaptive immune responses [120] (Figure 2).

The gut microbiota constantly primes the innate immune cells. Interestingly, macrophages but not DCs from antibiotic-treated mice exhibit impaired responsiveness to type 1/2 IFNs and reduced adaptive immunity stimulation of adaptive immunity [151]. These macrophages showed a reduced expression of genes associated with IFN activation and antiviral immunity, resulting in the exacerbation of lymphocytic choriomeningitis virus (LCMV) infection. Not only adaptive immunity-related cells but also effector innate immune cells, such as natural killer (NK) cells, are primed by type 1 IFN and IL-15 secreted from nonmucosal mononuclear phagocytes (DCs and macrophages); of note, mononuclear phagocytes from GF mice failed to induce the expression of type 1 IFN genes [152,153]. Although the PRR-mediated nuclear translocation of the transcription factors NF-κB and IRF3 did not change in mononuclear phagocytes of GF mice, their binding to their respective cytokine gene promoters was compromised, which correlated with the absence of activating histone marks. Therefore, gut microbiota plays a crucial role in the regulation of innate immune cells to secrete IFNs, and consequently in adaptive immunity.

However, not many reports have disclosed the microbial derivatives affecting innate immunity and the secretion of IFN. This said, recently a study revealed that the lipooligosaccharide (LOS) domain of polysaccharide A (PSA), derived from *B. fragilis*, contributes to the production of type 1 IFN (IFN-β) through TLR4-TRIF signaling in colonic but not small intestinal DCs [154]. IFN-β is functionally important for the priming of antiviral responses and the resulting resistance to vesicular stomatitis virus infection. Additionally, the microbial metabolite desaminotyrosine, which is produced by *Clostridium orbiscindens* through flavonoid metabolism, was shown to enhance type 1 IFN production and promote protection from influenza virus infection [155]; briefly, desaminotyrosine induced the amplification of type 1 IFN through IFNαR and the downstream of STAT1 in macrophages. Furthermore, microbe-derived SCFAs were shown to promote antibody production and the differentiation of B cells into plasma cells via the alteration of metabolic sensors and gene expression [156]. Particularly, butyrate plays a crucial role in the context of antimicrobial capacity in macrophages and memory potential in activated CD8^+^ T cells [157,158]. In macrophages, butyrate acts as a histone deacetylase inhibitor (HDACi), enhancing histone H3 acetylation, the downregulation of glycolysis, and the consequent reduction in the activity of mTOR kinase, resulting in enhanced antimicrobial activity. mTOR is a key regulator of autophagy and autophagy-related processes [159]. Interestingly, the expression of microtubule-associated protein 1 light chain 3 alpha (LC3) increased in butyrate-treated macrophages due to mTOR inhibition during *Salmonella* infection. Moreover, butyrate exposure was shown to promote lysosomal and antimicrobial gene expression in macrophages through HDACi activity. Although there is some controversy, butyrate was proposed to inversely upregulate glycolysis and uncouple the TCA cycle from glycolysis in antigen-activated CD8^+^ T cells, allowing the preferential fueling of oxidative phosphorylation via sustained glutamine utilization and fatty acid catabolism; this cellular metabolism change induces activated CD8^+^ T cells into memory cells. Furthermore, it was confirmed that transferring antigen-activated CD8^+^ T cells into GF mice resulted in their impaired differentiation into memory CD8^+^ T cells, suggesting that gut microbiota-derived butyrate is required to immune memory generation.

Overall, the above evidence clearly supports the notion that gut microbes and their derivatives regulate the number, differentiation, and function of host immune cells, and play a key role in the protection against pathogen infections.

## 6. Concluding Remarks

Here, we have reviewed the immunological mechanisms of action of commonly used vaccines and adjuvants. Furthermore, we have also discussed the gut microbiota as one of the factors influencing the efficacy of vaccines and adjuvants, and conclude that the stimulation by gut microbes is required for innate immune cells to mount optimal immune responses. In fact, gut microbiota-derived stimulation impacts not only local but also systemic immune responses to vaccination, suggesting that modulating the gut microbiota might be a new approach to improve vaccine efficacy. We also highlight that the intervention of prebiotic- and probiotic-related interventions have the potential to enhance the vaccines (particularly against influenza virus).

The gut microbiota is a very huge, diverse, and complex community, affected by host immunity. Thus, investigating the relationships between the gut microbiota and host immunity is essential to understand if it is possible to improve the efficacy vaccines (or adjuvants) via interventions targeting the gut microbiota. Comprehensive omics analyses might help us to investigate such relationships. Moreover, it is important to understand not only phenotypic alterations (e.g., the changes in the composition of the gut microbiota or in the levels of microbial-derived metabolites), but also the mechanisms behind the impact on host immunity. Detailed analysis of the effect of particular bacterial species in vaccine responses will give rise to the development of new strategies for the improvement of the efficacy of vaccines and adjuvants.

## Figures and Tables

**Table 1:** Current vaccines against respiratory tract diseases and their key protective mechanisms.

Diseases	Pathogenic Bacteria	Current Vaccine (Components)	Key Immune Responses for Protection
Tuberculosis	*Mycobacterium tuberculosis*	BCG vaccine (live-attenuated *M. bovis*)	・IFN-γ^+^ Th1 and IL-17^+^ Th17 cells ・Activating phagocytes and cytotoxic T cells
Pneumococcal pneumonia	*Streptococcus pneumoniae*	PPSV23 (23-valent polysaccharides only) PCV13 (carrier protein-conjugated 13-valent polysaccharides)	・Systemic IgG and mucosal sIgA production ・IL-17^+^ Th17 cells ・Elimination by monocytes and macrophages recruited by IL-17-induced chemokines expression
Influenza virus infection	Influenza virus	Live-attenuated or inactivated (split or subunit) influenza vaccine (influenza virus A: H1N1, H3N2) (influenza virus B: 1 or 2 strains)	・Systemic IgG and mucosal sIgA secretion against HA and NA of influenza virus ・Inducing CD8^+^ T_RM_ cells in nasal and lung
